# Well-balanced immune response and protective efficacy induced by a single dose of live attenuated tetravalent dengue vaccine (KD-382) in monkeys

**DOI:** 10.1016/j.heliyon.2020.e04506

**Published:** 2020-07-28

**Authors:** Masaya Yoshimura, Yasuhiko Shinmura, Shota Takagi, Kazuhisa Kameyama, Kengo Sonoda, Fusataka Koide, Sutee Yoksan, Kazuhiko Kimachi

**Affiliations:** a KM Biologics Co., Ltd., 1-6-1 Okubo, Kita-ku, Kumamoto-shi, Kumamoto 860-8568, Japan; b Southern Research, 431 Aviation Way, Frederick, MD 21701, USA; c Center for Vaccine Development, Institute of Molecular Biosciences, Mahidol University, 25/25 Phuttamonthon 4 Road, Salaya, Nakhon Pathom 73170, Thailand

**Keywords:** Microbiology, Virology, Antibody, Immune response, Vaccination, Vaccines, Viral disease, Live attenuated tetravalent dengue vaccine, Protective efficacy, KD-382

## Abstract

One of the challenges developing a live attenuated tetravalent dengue vaccine (TDV) is to overcome the presumed viral interference that may be preventing the induction of a balanced immune response to all 4 serotypes of the dengue virus (DENV1–4). Our live attenuated TDV candidate was developed from wild-type (wt) parental strains (DENV1/03135, DENV2/99345, DENV3/16562, and DENV4/1036, respectively) using a classical host range mutation strategy: the same strategy used for the approved live attenuated smallpox, polio, and MMR vaccines. Our vaccine candidate is expected to mimic natural dengue virus infection, as it provides all the components of dengue virus, including both structural and nonstructural proteins. Therefore, induction of more solid and comprehensive immune responses against pathogenic dengue viruses is also expected. In this study, we evaluated the neutralizing antibody responses for each serotype induced by a single subcutaneous administration of 6 formulations, which were composed of different combinations of vaccine strains and were all of different dosages. These formulations were tested in dengue-naïve cynomolgus macaques. As a result, regardless of the TDV formulation, all the monkeys immunized with TDVs seroconverted to all the 4 serotypes at day 30. Next, we evaluated protection ability of the selected formulations of TDV candidate, no RNAemia was detected from any of the immunized monkeys upon s.c. challenge with wtDENV. The findings of this non-human primate study indicate that our vaccine candidate is very promising; it can be further evaluated for safety and efficacy in human clinical studies.

## 1 Introduction

Dengue virus (DENV) infection is endemic worldwide, particularly in tropical and subtropical regions, with 40% of the world's population at risk of infection [1]. DENVs have 4 serotypes (DENV1–4), which occasionally cause serious symptoms, such as dengue hemorrhagic fever (DHF) and dengue shock syndrome [2]. These serious symptoms are related to cross-reactive heterotypic antibodies induced by the primary infection, which acts as a risk factor, and the symptoms occur upon the secondary infection with another serotype virus. This phenomenon is known as antibody-dependent enhancement (ADE) [3, 4]. If a dengue vaccine induces unbalanced and/or non-neutralizing antibodies, it may introduce the risk of ADE like natural secondary infection cases. Therefore, a tetravalent dengue vaccine (TDV) capable of inducing a balanced antibody levels against all 4 DENV serotypes should be developed for both efficacy and safety purposes.

Dengue vaccines have been developed worldwide and are mostly live attenuated [5]. For live vaccines, it is necessary to attenuate the pathogenicity until they are tolerated while inducing a sufficient immune response. Generally, live vaccines are produced using host range mutants attenuated by their passage through different animal cells other than the natural host. Dengue vaccine was conventionally developed using this method from 1980 to 2000. Because the DENV has 4 serotypes, vaccine strains with acceptable safety and immunogenicity for each serotype need to be established. However, interference among different viruses in combined live vaccines is a common phenomenon [6, 7, 8]; therefore, a detailed evaluation in a combined formulation covering both immunogenicity and safety aspects is crucial. For TDV containing all 4 serotypes (DENV1–4), *in vivo* interference among each strain is also one of the most prominent concerns. Indeed, in the development of a live attenuated TDV conducted by Mahidol University (Mahidol TDV) [9, 10], no problems were faced with the monovalent, bivalent, or trivalent vaccine conditions of DENV1, 2, and 4, confirming the acceptable safety and well-balanced induction of neutralizing antibodies. However, when DENV3 was added to create a TDV, side reactions that could be attributed to the under attenuation of DENV3 occurred along with a decreased neutralizing antibody response against DENV1, 2, 4. This is thought to be due to interference between DENV3 and other viruses. Thus, this alleged interference has impeded the development of an attenuated dengue vaccine based on the host range variants for all four serotypes.

In the meantime, the focus of mainstream research and development of a live attenuated dengue vaccine has shifted to a generation of vaccine strains created by chimera technology; this technique first appeared in the 1990s, when gene recombination technology became applicable to live vector vaccines. CYD-DENV (Dengvaxia®, developed as a chimeric virus with yellow fever vaccine strain YF-17D) was first approved in Mexico in 2015 [1] and has since been approved in 20 countries worldwide [11]. However, post-marketing surveillance (Phase 3 follow-up) has shown that the vaccination of dengue-naïve children may exacerbate diseases caused by natural infection [12]. As a result, the Dengvaxia® can only be used for people who have been previously infected with the DENV and who are 9–45 years of age; this is due to the vaccine's low efficacy and the risk of infection among seronegative individuals [1, 13]. In a position paper, the World Health Organization (WHO) recommended pre-vaccination screening as a practical strategy to avoid the problem among seronegative individuals [13]. Under careful conditions, the Dengvaxia® can be valuable as a first-generation dengue vaccine. Other genetically modified chimeric vaccines have also been developed. TAK003 is consisting of a combination of full-length DENV2 and chimeric viruses (DENV1, 3, and 4) with a DENV2 backbone that is under development by Takeda [14]. An article on the details of Phase 3 results was published [15], and it shows an overall vaccine efficacy of more than 80%. However, the efficacy varies according to individual serotype, and the vaccine efficacy against DENV3 and 4 were approximately 50%. NIH/Butantan vaccines, TV003/005, are a combination of deletion mutants (DENV1, 3, and 4) lacking the 3′-UTR 30 and/or 31 bases of each serotype and a chimeric virus (DENV2) with a DENV4 backbone [16]. Although there are no monkey studies similar to the current studies, both TV003 and TV005 have achieved a tetravalent seroconversion rate of approximately 90% in a human clinical study [17]. In order to develop a much more effective dengue vaccine, we consider it important that the dengue vaccine can induce neutralizing antibodies (Nabs) with no single serotype being induced in greater quantities than the other serotypes and no suppression of the other three serotypes (well-balanced), similar to TV003/005 [18,19]. At the same time, Dengvaxia® is a yellow fever virus-based chimeric vaccine that contains only part of the DENV's proteins: pre M and E proteins [20, 21]. Therefore, a lack of the other viral proteins may be responsible for the severe dengue cases in seronegative vaccinees; this is supported by the fact that no increase in severe dengue infections was observed although Mahidol TDV with both structural and non-structural DENV antigens induced an imbalanced immune response in human [22]. In order to develop a more effective dengue vaccine that can be used for any target population, we recognize that balanced responses in both humoral and cellular immunity are very important.

We consider classical host range variants that can provide all of the DENV components, including nonstructural proteins as antigens, to be the most appropriate approach to establishing a realistic vaccine. Guided by this concept, we generated a new set of attenuated TDVs that are a modification of the Mahidol TDV. In this development, we aimed to establish a TDV that can provide the following features through single-dose administration: 1) Achievement of seroconversion of Nabs for all four serotypes in a short period of time; 2) Induction of a balanced NAb for all four serotypes; 3) Long-lasting antibody seroconversion status for all serotypes; 4) Low viremic status after administration of the vaccine; 5) Immune responses against the nonstructural viral proteins; and 6) Protection against viremia following live DENV challenge.

In this study, we evaluated candidate TDV formulations in cynomolgus monkeys for our selection of a combination that can be further evaluated in non-clinical and clinical studies based on the above points 1, 2, 4, 6. In addition, as other important points, immune status after challenge were evaluated.

## 2 Materials and Methods

### 2.1 Vaccine

To prepare each monovalent candidate vaccine seed virus was subcultured in Vero cells and then concentrated. The live TDVs used in this study were formulated by mixing monovalent virus solutions of the 4 serotypes in KM Biologics Co., Ltd. (Kumamoto, Japan). The parental DENVs, DENV1 and DENV4, were isolated from dengue fever (DF) patients, and DENV2 and DENV3 were isolated from DHF patients. DENV1, DENV2, and DENV4 parental viruses were attenuated by serial passage in certified primary dog kidney (PDK) cells, purchased from the Netherlands, whereas the DENV3 parental virus was attenuated by serial passage in PDK cells after passaging on primary green monkey kidney (PGMK) cells. DENV3 in the Mahidol TDV, which is the predecessor of the vaccine candidate produced by KM Biologics, was only passaged in PGMK cells for attenuation [9]. It is considered that the DENV3 in the Mahidol TDV was not sufficiently attenuated. To improve this issue, the DENV3 strains in the current candidates were additionally passaged in PDK cells in the same way as DENV1, DENV2, and DENV4. We used the same serotype 4 that was involved in the Mahidol TDV; however, the number of passages was reduced from 48 to 45 or 40 in order to increase the immunogenicity. The DENV1 and 2 parental strains were different than those used in the Mahidol TDV. Details of the TDV strains and passage histories are shown in Table 1. Viral titrations were performed by a plaque assay or an immunostaining titration assay using Vero cells and serotype-specific monoclonal antibodies and were expressed as PFU/mL or FFU/mL, respectively.

**Table 1:** Details of TDV passage histories.

Vaccine strains used in the current study				
	DENV1	DENV2	DENV3	DENV4
Clinical isolates (parental strains)	03135 (7-year-old DF patient, Thailand, 2003)	99345 (10-year-old DHF patient, Thailand, 1999)	16562 (DHF patient, Philippines, 1964)	1036 (DF patient, Indonesia, 1976)
Vaccine candidate Strain A	PDK15V3	PDK25	PGMK30PDK4	PDK40
Vaccine candidate Strain B	Not applicable	PDK35	PGMK30PDK3	PDK45

Vaccine strains of the Mahidol TDV				

Parental strains	16007 (DHF patient, Thailand, 1964)	16681 (DHF patient, Thailand, 1964)	16562 (DHF patient, Philippines, 1964)	1036 (DF patient, Indonesia, 1976)
Vaccine strains	PDK13	PDK53	PGMK30FRhL3	PDK48

V: Vero cell; FRhL: Fetal Rhesus Lung cell. The name of each vaccine strain is derived from the cultured cell line followed by the passage number (e.g., the PDK15V3 strain was subcultured 15 times with PDK cells and 3 times with Vero cells).

### 2.2 Animal experiments

#### 2.2.1 [Study 1] Vaccine Candidate Virus Screening: Assessment of DENV1–4 With Different Passage Histories and Different Dose Combinations

As shown in Tables 1 and 2, multiple candidate virus sets with different passage histories and different dose combinations were evaluated in monkeys. Monkeys inoculated with 5 log_10_ or 3 log_10_ PFU of wild-type DENVs (wtDENV1–4) served as positive controls. Immune responses and viremia were investigated based on the schedule shown in Table 3.

**Table 2:** Experimental group of Study 1.

Group	Inoculum	Dose	N
1	wtDENV1	5 log_10_ PFU/animal	3
2	wtDENV2	5 log_10_ PFU/animal	3
3	wtDENV2	3 log_10_ PFU/animal	3
4	wtDENV3	5 log_10_ PFU/animal	3
5	wtDENV4	5 log_10_ PFU/animal	3
6	wtDENV4	5 log_10_ PFU/animal	3
7	TDV-AAAA	5, 5, 5, 5 log_10_ PFU/animal	3
8	TDV-AAAA	5, 3, 5, 3 log_10_ PFU/animal	3
9	TDV-AAAA	5, 3, 3, 3 log_10_ PFU/animal	3
10	TDV-AAAB	5, 5, 5, 5 log_10_ PFU/animal	3
11	TDV-ABAB	5, 5, 5, 5 log_10_ PFU/animal	3
12	TDV-ABBB	5, 5, 5, 5 log_10_ PFU/animal	3

The wtDENVs used for inoculations and challenges were parental clinical isolates subcultured with Vero cells several times prior to use.

**Table 3:** Schedule for study 1.

Day of study	0	1	2	3	4	6	8	10	14	30	60
Procedure											
Vaccination	✓										
Daily observation	All animals were monitored twice daily										
**Blood sampling:**											
qPCR viral load	✓	✓	✓	✓	✓	✓	✓	✓	✓		
PRNT	✓								✓	✓	✓
Euthanasia											✓

#### 2.2.2 [Study 2] Dose Selection Study: Assessment of Dose in Selected combination (5 log_10_, 4 log_10_, 3 log_10_ FFU)

A combination of the candidate strains selected in study 1 was further evaluated for its immunogenicity and protective efficacy with different doses. Animals were divided into three groups with three vaccine formulations, each with different doses (Table 4). These doses were high (all 5 log_10_ FFU/dose), moderate (all 4 log_10_ FFU/dose), low (all 3 log_10_ FFU/dose), and vehicle (as a negative control) and were administered on day 0, as described in Table 5. The immune responses and viremia (RNAemia) after the vaccine administration were evaluated according to the schedule shown in Table 5. For protection experiment, the monkeys were challenged with the wtDENVs of each serotype at 60 days post-vaccination; RNAemia was assessed by qPCR. The challenge method was a subcutaneous injection of 0.5 mL or 1.0 mL of the wtDENV in the left or right forearm. The wtDENVs used for challenges were parental clinical isolates subcultured with Vero cells several times prior to use.

**Table 4:** Design of study 2.

Group	Vaccination (Day 0)	Challenge (Day 60)		Monkeys
	Inoculum (log_10_ FFU/animal)	Virus	Dose (log_10_ FFU/animal)	N
1	TDV-AAAA (5, 5, 5, 5)	wtDENV1	5	6
		wtDENV2	5	6
		wtDENV3	6	6
		wtDENV4	5	6
2	TDV-AAAA (4, 4, 4, 4)	wtDENV1	5	6
		wtDENV2	5	6
		wtDENV3	6	6
		wtDENV4	5	6
3	TDV-AAAA (3, 3, 3, 3)	wtDENV1	5	6
		wtDENV2	5	6
		wtDENV3	6	6
		wtDENV4	5	6
4	Vehicle (N/A)	wtDENV1	5	3
		wtDENV2	5	3
		wtDENV3	6	3
		wtDENV4	5	3

**Table 5:** Schedule for study 2.

A. Vaccination phase											
Study day	0	1	2	3	4	6	8	10	14	30	60
Procedure											
Vaccination	✓										
Daily observation	All animals were monitored twice daily										
**Blood sampling**											
qPCR viral load	✓	✓	✓	✓	✓	✓	✓	✓	✓		✓
PRNT	✓								✓	✓	✓

B. Post-challenge phase										
Study day	60	61	62	63	64	66	68	70	74	90
Procedure										
Challenge	✓									
Daily observation	All animals were monitored twice daily									
**Blood collections**										
qPCR Viral Load	✓	✓	✓	✓	✓	✓	✓	✓	✓	
PRNT	✓								✓	✓
Euthanasia										✓

### 2.3 Animals (age, sex, number, etc.)

Cynomolgus monkeys were used. A total of 36 monkeys (18 males and 18 females) aged 2–5 years (2.9–6.3 kg) were used in study 1. In study 2, 84 monkeys (42 males and 42 females) aged 3–5 years (2.0–6.0 kg) were used. In both studies, all monkeys were randomized into respective groups according to sex and weight using Provantis® (Instem™ LSS Ltd., Staffordshire, UK) as outlined in Table 2 and Table 4, respectively.

### 2.4 Facilities management and animal ethical aspects

Animal care was in compliance with the Guide for the Care and Use of Laboratory Animals, 8th Edition (Institute of Animal Resources, Commission on Life Sciences, National Research Council; National Academy press; Washington, DC; 2011), and the U.S. Department of Agriculture through the Animal Welfare Act (Public Law 99–198). Southern Research Institute is fully accredited by the Association for (AAALAC)Assessment and Accreditation of Laboratory Animal Care International.

The study protocols were reviewed and approved by the IACUC (Institutional Animal Care and Use Committee) at Southern Research Institute or additionally KM Biologics Co., Ltd.

### 2.5 Viral load measurement assay

The viral load was determined using RT-qPCR to detect the DENV genomes in serum samples. Primers and probes were designed with minor modifications according to previous studies [23, 24], and synthesized by Operon (Huntsville, AL) or Eurofins MWG Operation LLC (Huntsville, AL). In study 1, infectious virus titers in the serum were determined using a plaque assay. The samples used for the plaque assay were sera at the first- and second-highest viral peaks in the RT-qPCR.

### 2.6 NAb titration assay

An immunospot plaque 50% reduction neutralization test (PRNT_50_) assay against wtDENV1–4 virus was performed to determine the serum NAb titers. Mixed input viruses and fourfold serially diluted serum (1:10 initial dilution) samples were incubated for 1 h for neutralization and inoculated with 100 μL into 96-well plates seeded with pre-prepared LLC-MK2 cells. After 1 h of adsorption, an overlay medium was added to the plates and incubated overnight under 5% CO_2_ conditions. The next day, the plates were stained using serotype-specific or broad-spectrum dengue antibodies and goat anti-mouse IgG (H + L) HRP-conjugated secondary antibodies. A TrueBlue peroxidase substrate was added to the plates, and the spots were analyzed by a BioSpot scanner.

### 2.7 Deep sequencing and amino acid substitution analyses

Deep sequencing of both parental viruses and vaccine seed viruses of candidate A was conducted using Illumina sequencers (Illumina, San Diego, CA). For the parental viruses, RNA was extracted from the culture fluid using QIAamp Viral RNA Mini Kit (QIAGEN, Venlo, the Netherlands) and submitted to an external sequencing service provider (Hokkaido system science Co. Ltd., Hokkaido, Japan) for preparation of RNA-seq libraries and sequencing on the Illumina HiSeq platform, which generated 101 bp paired-end raw reads data sets in the FASTQ format. For the vaccine seed viruses of candidate A, RNA was extracted from the UF-cartridge concentrated, Benzonase-treated (Merck, Darmstadt, Germany) culture fluid using ZR Viral RNA kit (Zymo Research, Irvine, CA). Sequence libraries were prepared using NEBNext Ultra RNA Library Prep Kit for Illumina (New England Biolabs, Ipswich, MA), quality checked with Qubit Fluorometer (Thermo Fisher Scientific, Waltham, MA) and Agilent 2100 Bioanalyzer (Agilent Technologies, Santa Clara, CA), and sequenced on the Illumina MiSeq platform, which generated 151 bp paired-end raw reads data sets in the FASTQ format.

The data sets were processed by the following pipeline to obtain comparison data between the parental and vaccine seed viruses of each serotype. First, the data sets were cleaned up by trimming 1nt from the 3 end of reads and removing reads containing ambiguous nucleotides using FASTX Toolkit 0.0.14 (http://hannonlab.cshl.edu/fastx_toolkit/index.html), removing reads containing adapter sequences using Cutadapt 1.6 [25], retaining reads of which the base call qualities of more than 80 % of the bases were higher than a threshold of Q20, and trimming bases with qualities lower than Q20 from 3ʹ end of reads using FAXTX Toolkit 0.0.14 and PRINSEQ-lite 0.20.4 [26], and extracting remaining paired reads. Second, the cleaned-up data sets were mapped to in-house viral reference genomes using Burrows-Wheeler Aligner 0.7.10 with the MEM algorithm [27] and pileup files were generated using SAMtools 1.1 [28]. Finally, the pileup files were processed to generate amino acid composition data for a better biological interpretation of the vaccine seed viruses, and variants were called if there were changes of 10% or more in frequencies of amino acid residues at an amino acid position between the parental and vaccine seed viruses using in-house Perl scripts. A cut off value of 10% in amino acid frequencies was applied to exclude minor variants. The aforementioned in-house viral reference genomes were previously generated by fine-tuning reference genomes on GenBank (Accession: AY732479.1, FJ744714.1, JN697379.1, KF907503.1), which were closest to the seed viruses, with nucleotide substitutions as per sequencing data of vaccine seed viruses.

## 3 Results

### 3.1 [Study 1]

#### 3.1.1 All TDV formulations induced tetravalent Nab responses

**Figure 1:**
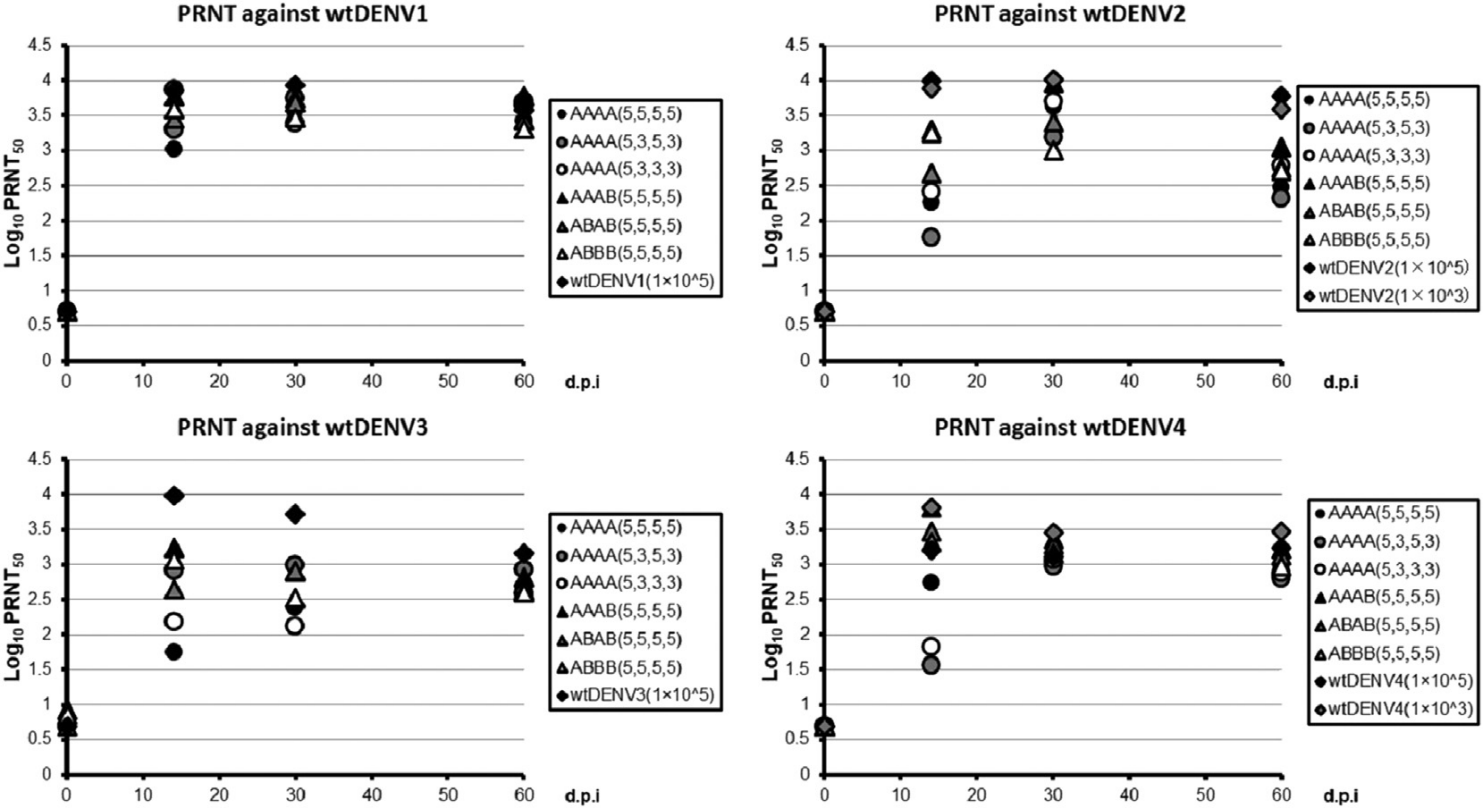
**All candidates elicited NAb responses**. A PRNT assay against the wt parental DENV1–4 virus was conducted in serum samples collected prior to administration on Day 0 and on days 14, 30, and 60 after administration. For the calculation of the GMT, data reported as “<10” were converted to “5” and data reported as “≥10,240” were converted to “10,240”.

NAbs were positive for all four serotypes at day 14, except for one individual in group 8. The DENV specific log_10_ geometric mean titer (GMT) of the NAb at day 14 varied from 3.02 to 3.87 (DENV1), 1.76 to 3.30 (DENV2), 1.75 to 3.23 (DENV3), and 1.56 to 3.81 (DENV4). Notably, the NAb titers against DENV1 were robust. On day 30, an increase in DENV2 and DENV4 NAb titers was observed in 5 of 6 and 3 of 6 TDV groups, respectively. On day 30, the log_10_ GMT to each serotype ranged from 3.40 to 3.75 (DENV1), 3.01 to 3.96 (DENV2), 2.11 to 2.99 (DENV3), and 2.98 to 3.36 (DENV4). On day 60, the log_10_ GMT of the TDV group to each serotype ranged from 3.32 to 3.78 (DENV1), to 2.33 to 3.05 (DENV2), 2.59 to 2.92 (DENV3), and 2.80 to 3.22 (DENV4). The antibody titers were more likely to converge on day 60 rather than days 14 and/or 30 and this phenomenon was especially strong in Type 3. No dose dependency (5 log_10_ or 3 log_10_ PFU) on the immune response was observed. At the same time, there was no difference in the NAb response induced by the different TDV formulations, regardless of whether candidates A or B were included (Figure 1).

#### 3.1.2 RNAemia caused by the TDV candidates were lower than those caused by wtDENVs

The monkeys in groups 1–6 were inoculated with 1 × 10^3^ or 1 × 10^5^ PFU of wtDENV1–4 by a single subcutaneous injection. DENV1 RNAemia (group 1) was detectable in all three monkeys on days 1 and 2, with individual viral loads ranging from 1.4 × 10^4^ to 5.9 × 10^5^ GEq/mL and 7.4 × 10^3^ to 3.3 × 10^4^ GEq/mL, respectively. DENV2 RNAemia in group 2 (1 × 10^5^ PFU) was detectable in two of three monkeys on day 1, whereas none of the lower dose inoculation (1 × 10^3^ PFU) monkeys in group 3 exhibited RNAemia. In regard to wtDENV3 (group 4), only transient and sporadic viremia was observed. The robust viremia of wtDENV4 was detected on day 1 in all three animals in group 5 (1 × 10^5^ PFU), with individual titers ranging from 1.5 × 10^4^ to 3.5 × 10^5^ GEq/mL.

**Figure 2:**
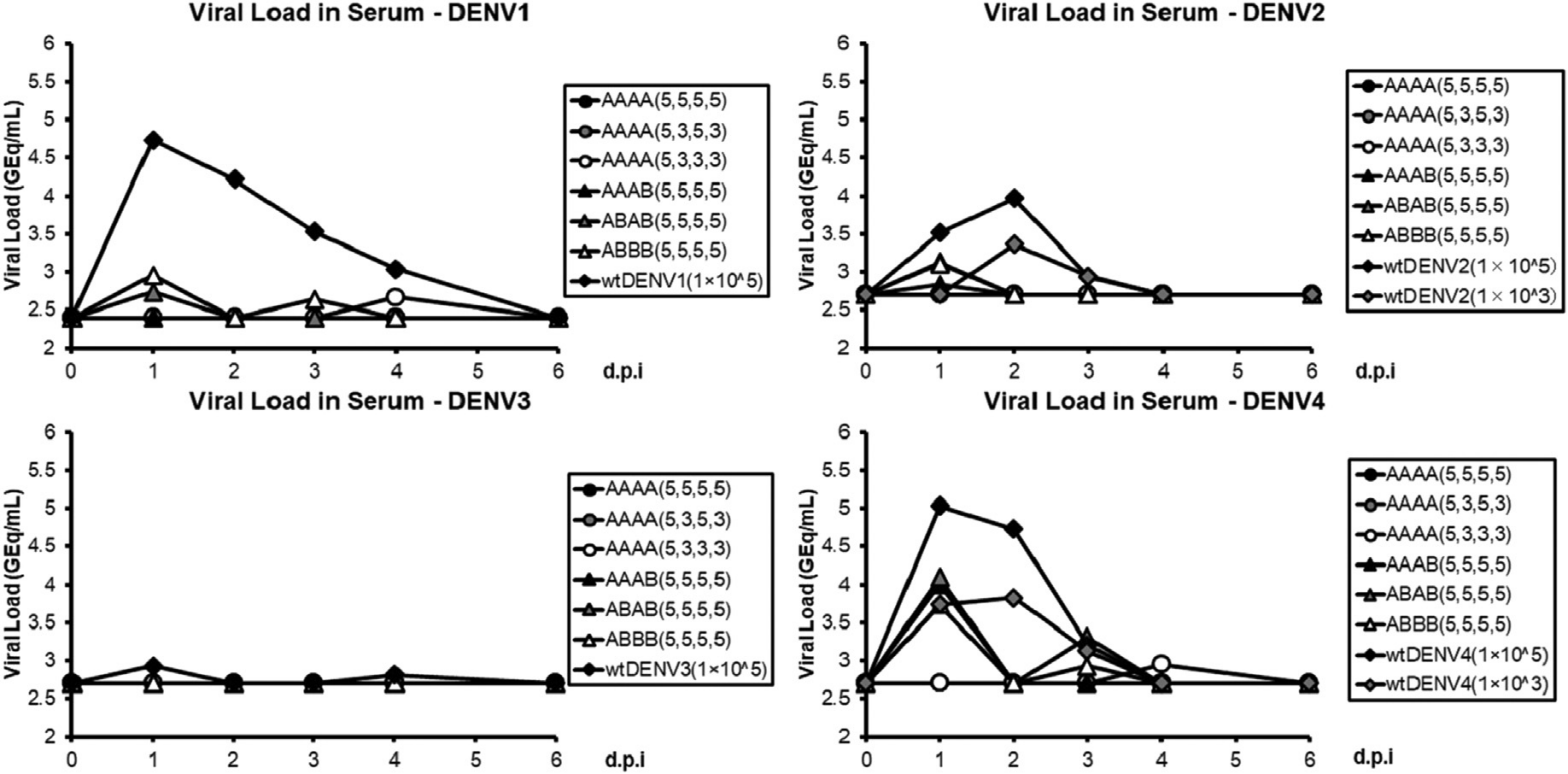
**The viral loads (GMT) in monkeys immunized with TDV candidates were lower than those of the monkeys inoculated with wtDENVs**. The viral load (genome copies/mL) was measured by RT-qPCR in serum samples collected prior to administration on Day 0 and on Days 1, 2, 3, 4, 6, 8, 10, and 14 after administration. Although not shown in the figure, the day 8, 10, and 14 viral loads were reported as below LLOQ for all groups.

All of the TDV candidate immunized monkeys (groups 7–12) had reduced virus loads when compared with the single corresponding wtDENV inoculated monkeys (groups 1–6), and none of the TDV-immunized macaques developed any detectable DENV3 RNAemia. The RNAemia in both Group 7 TDV-AAAA (5, 5, 5, 5) and group 8 TDV-AAAA (5, 3, 5, 3) monkeys was below the Lower Limit of Quantification (LLOQ) (<500 GEq/mL for DENV1 and <1,000 GEq/mL for DENV2–4) post-vaccination (day 0–14). One monkey in Group 9 TDV-AAAA (5, 3, 3, 3) developed transient RNAemia on day 4 for DENV1 and DENV4, with viremia titers of 1.6 × 10^3^ and 2.8 × 10^3^ GEq/mL, respectively. Group 10 TDV-AAAB (5, 5, 5, 5), which had a candidate B strain of DENV4, showed a high viral load of DENV4 in all three monkeys, with titers ranging from 3.2 × 10^3^ to 3.2 × 10^4^ GEq/mL on day 1. In Group 11 TDV-ABAB (5, 5, 5, 5), which had an additional candidate B strain of DENV2, all three monkeys developed DENV2 and DENV4 RNAemia on day 1, with individual titers ranging from 1.0 × 10^3^ to 1.8 × 10^3^ GEq/mL and 9.5 × 10^3^ to 2.0 × 10^4^ GEq/mL, respectively. A transient DENV4 titer was detected on day 3 in two of three monkeys with titers of 2.3 × 10^3^ and 7.5 × 10^3^ GEq/mL. Additionally, for this group, a transient DENV1 viremia was detected on day 1 in two of three monkeys with titers of 7.5 × 10^2^ and 8.7 × 10^2^ GEq/mL. Group 12 TDV-ABBB (5, 5, 5, 5), with vaccine candidate B strains with the exception of DENV1, had an RNAemia profile similar to group 11. Overall, the inclusion of DENV1, 2, and 4 vaccine candidate B strains in TDV formulations (groups 10–12) led to a tendency for a higher RNAemia of DENV4 than those in groups 7–9 with the candidate A strain; however, the DENV4 vaccine RNAemia was sporadically detected on days 1, 3, and 4, and the peak viral load was approximately tenfold lower than that of wtDENV4 (Tables 6, 7, 8, 9, and 10, Figure 2).

**Table 6:** Daily RNAemias in susceptible cynomolgus monkeys given monotypic wild type DENV 1–4 viruses.

Group	Inoculum	Viral load in serum (log_10_ in genome copies/mL)													
		Day 0		Day 1		Day 2		Day 3		Day 4		Day 6		Day 8	
1	wtDENV1 (5 log_10_ PFU)	<2.40	<2.40	4.74	4.30	4.22	4.51	3.53	<2.40	3.04	<2.40	<2.40	<2.40	<2.40	<2.40
			<2.40		5.77		3.87		3.84		3.16		<2.40		<2.40
			<2.40		4.13		4.28		4.34		3.57		<2.40		<2.40
2	wtDENV2 (5 log_10_ PFU)	<2.70	<2.70	3.52	3.77	3.97	3.56	2.94	<2.70	<2.70	<2.70	<2.70	<2.70	<2.70	<2.70
			<2.70		<2.70		3.87		3.07		<2.70		<2.70		<2.70
			<2.70		4.06		4.49		3.04		<2.70		<2.70		<2.70
3	wtDENV2 (3 log_10_ PFU)	<2.70	<2.70	<2.70	<2.70	3.36	3.50	2.95	<2.70	<2.70	<2.70	<2.70	<2.70	<2.70	<2.70
			<2.70		<2.70		<2.70		<2.70		<2.70		<2.70		<2.70
			<2.70		<2.70		3.89		3.44		<2.70		<2.70		<2.70
4	wtDENV3 (5 log_10_ PFU)	<2.70	<2.70	2.93	3.38	<2.70	<2.70	<2.70	<2.70	2.81	<2.70	<2.70	<2.70	<2.70	<2.70
			<2.70		<2.70		<2.70		<2.70		<2.70		<2.70		<2.70
			<2.70		<2.70		<2.70		<2.70		3.03		<2.70		<2.70
5	wtDENV4 (5 log_10_ PFU)	<2.70	<2.70	5.03	5.37	4.73	3.84	3.20	3.44	<2.70	<2.70	<2.70	<2.70	<2.70	<2.70
			<2.70		4.19		4.89		<2.70		<2.70		<2.70		<2.70
			<2.70		5.54		5.45		3.47		<2.70		<2.70		<2.70
6	wtDENV4 (3 log_10_ PFU)	<2.70	<2.70	3.73	4.51	3.83	4.19	3.13	3.25	<2.70	<2.70	<2.70	<2.70	<2.70	<2.70
			<2.70		3.99		3.81		3.44		<2.70		<2.70		<2.70
			<2.70		<2.70		3.48		<2.70		<2.70		<2.70		<2.70

**Table 7:** Daily DENV 1 RNAemias in susceptible cynomolgus monkeys given different candidate tetravalent DENV vaccines.

Group	Inoculum	DENV1 viral load in serum (log_10_ in genome copies/mL)							
		Day 1		Day 2		Day 3		Day 4	
7	TDV-AAAA (5, 5, 5, 5)	<2.40	<2.40	<2.40	<2.40	<2.40	<2.40	<2.40	<2.40
			<2.40		<2.40		<2.40		<2.40
			<2.40		<2.40		<2.40		<2.40
8	TDV-AAAA (5, 3, 5, 3)	<2.40	<2.40	<2.40	<2.40	<2.40	<2.40	<2.40	<2.40
			<2.40		<2.40		<2.40		<2.40
			<2.40		<2.40		<2.40		<2.40
9	TDV-AAAA (5, 3, 3, 3)	<2.40	<2.40	<2.40	<2.40	<2.40	<2.40	2.67	<2.40
			<2.40		<2.40		<2.40		<2.40
			<2.40		<2.40		<2.40		3.21
10	TDV-AAAB (5, 5, 5, 5)	<2.40	<2.40	<2.40	<2.40	<2.40	<2.40	<2.40	<2.40
			<2.40		<2.40		<2.40		<2.40
			<2.40		<2.40		<2.40		<2.40
11	TDV-ABAB (5, 5, 5, 5)	2.74	2.87	<2.40	<2.40	<2.40	<2.40	<2.40	<2.40
			<2.40		<2.40		<2.40		<2.40
			2.94		<2.40		<2.40		<2.40
12	TDV-ABBB (5, 5, 5, 5)	2.96	3.49	<2.40	<2.40	2.64	<2.40	<2.40	<2.40
			3.00		<2.40		3.12		<2.40
			<2.40		<2.40		<2.40		<2.40

**Table 8:** Daily DENV 2 RNAemias in susceptible cynomolgus monkeys given different candidate tetravalent DENV vaccines.

Group	Inoculum	DENV2 viral load in serum (log_10_ in genome copies/mL)							
		Day 1		Day 2		Day 3		Day 4	
7	TDV-AAAA (5, 5, 5, 5)	<2.70	<2.70	<2.70	<2.70	<2.70	<2.70	<2.70	<2.70
			<2.70		<2.70		<2.70		<2.70
			<2.70		<2.70		<2.70		<2.70
8	TDV-AAAA (5, 3, 5, 3)	<2.70	<2.70	<2.70	<2.70	<2.70	<2.70	<2.70	<2.70
			<2.70		<2.70		<2.70		<2.70
			<2.70		<2.70		<2.70		<2.70
9	TDV-AAAA (5, 3, 3, 3)	<2.70	<2.70	<2.70	<2.70	<2.70	<2.70	<2.70	<2.70
			<2.70		<2.70		<2.70		<2.70
			<2.70		<2.70		<2.70		<2.70
10	TDV-AAAB (5, 5, 5, 5)	2.83	<2.70	<2.70	<2.70	<2.70	<2.70	<2.70	<2.70
			3.09		<2.70		<2.70		<2.70
			<2.70		<2.70		<2.70		<2.70
11	TDV-ABAB (5, 5, 5, 5)	3.13	3.24	<2.70	<2.70	<2.70	<2.70	<2.70	<2.70
			3.00		<2.70		<2.70		<2.70
			3.13		<2.70		<2.70		<2.70
12	TDV-ABBB (5, 5, 5, 5)	3.11	3.48	<2.70	<2.70	<2.70	<2.70	<2.70	<2.70
			3.15		<2.70		<2.70		<2.70
			<2.70		<2.70		<2.70		<2.70

**Table 9:** Daily DENV 3 RNAemias in susceptible cynomolgus monkeys given different candidate tetravalent DENV vaccines.

Group	Inoculum	DENV3 viral load in serum (log_10_ in genome copies/mL)							
		Day 1		Day 2		Day 3		Day 4	
7	TDV-AAAA (5, 5, 5, 5)	<2.70	<2.70	<2.70	<2.70	<2.70	<2.70	<2.70	<2.70
			<2.70		<2.70		<2.70		<2.70
			<2.70		<2.70		<2.70		<2.70
8	TDV-AAAA (5, 3, 5, 3)	<2.70	<2.70	<2.70	<2.70	<2.70	<2.70	<2.70	<2.70
			<2.70		<2.70		<2.70		<2.70
			<2.70		<2.70		<2.70		<2.70
9	TDV-AAAA (5, 3, 3, 3)	<2.70	<2.70	<2.70	<2.70	<2.70	<2.70	<2.70	<2.70
			<2.70		<2.70		<2.70		<2.70
			<2.70		<2.70		<2.70		<2.70
10	TDV-AAAB (5, 5, 5, 5)	<2.70	<2.70	<2.70	<2.70	<2.70	<2.70	<2.70	<2.70
			<2.70		<2.70		<2.70		<2.70
			<2.70		<2.70		<2.70		<2.70
11	TDV-ABAB (5, 5, 5, 5)	<2.70	<2.70	<2.70	<2.70	<2.70	<2.70	<2.70	<2.70
			<2.70		<2.70		<2.70		<2.70
			<2.70		<2.70		<2.70		<2.70
12	TDV-ABBB (5, 5, 5, 5)	<2.70	<2.70	<2.70	<2.70	<2.70	<2.70	<2.70	<2.70
			<2.70		<2.70		<2.70		<2.70
			<2.70		<2.70		<2.70		<2.70

**Table 10:** Daily DENV 4 RNAemias in susceptible cynomolgus monkeys given different candidate tetravalent DENV vaccines.

Group	Inoculum	DENV4 viral load in serum (log_10_ in genome copies/mL)							
		Day 1		Day 2		Day 3		Day 4	
7	TDV-AAAA (5, 5, 5, 5)	<2.70	<2.70	<2.70	<2.70	<2.70	<2.70	<2.70	<2.70
			<2.70		<2.70		<2.70		<2.70
			<2.70		<2.70		<2.70		<2.70
8	TDV-AAAA (5, 3, 5, 3)	<2.70	<2.70	<2.70	<2.70	<2.70	<2.70	<2.70	<2.70
			<2.70		<2.70		<2.70		<2.70
			<2.70		<2.70		<2.70		<2.70
9	TDV-AAAA (5, 3, 3, 3)	<2.70	<2.70	<2.70	<2.70	<2.70	<2.70	2.95	<2.70
			<2.70		<2.70		<2.70		<2.70
			<2.70		<2.70		<2.70		3.45
10	TDV-AAAB (5, 5, 5, 5)	4.00	4.01	<2.70	<2.70	<2.70	<2.70	<2.70	<2.70
			4.50		<2.70		<2.70		<2.70
			3.50		<2.70		<2.70		<2.70
11	TDV-ABAB (5, 5, 5, 5)	4.09	4.01	<2.70	<2.70	3.31	<2.70	<2.70	<2.70
			3.98		<2.70		3.35		<2.70
			4.29		<2.70		3.88		<2.70
12	TDV-ABBB (5, 5, 5, 5)	3.78	4.26	<2.70	<2.70	2.93	<2.70	<2.70	<2.70
			4.25		<2.70		3.39		<2.70
			<2.70		<2.70		<2.70		<2.70

In the above tables (Tables 6, 7, 8, 9, and 10), the numbers on the left indicate GMT value and the numbers on the right indicate the RNAemia values for each individual. The LLOQ was 500 GEq/mL for DENV1 and 1000 GEq/mL for DENV2–4. For the calculation of GMT, data reported as “<500” were converted to “250,” and data reported as “<1,000” were converted to “500.”

#### 3.1.3 Among all the candidates, the lowest viremia level was observed in TDV-AAAA (5, 5, 5, 5)

Serum samples from animals inoculated with a wt parental virus (groups 1–6) and from TDV vaccinated animals (groups 7–12) were tested by a standard plaque assay for infectious virus particles (PFU/mL). Serum samples yielding the first- and second-highest RNAemia samples were selected. Viral loads in PFU/mL are summarized in Table 11. Infectious wtDENV1 virus (group 1) was detectable in all three monkeys at the first- and second-highest viremia peaks, yielding 394.6 PFU/mL (GMT) and 405.8 PFU/mL (GMT), respectively. A viral load was detectable in all six monkeys from groups 5 and 6, who received 1 × 10^5^ and 1 × 10^3^ PFU of wtDENV4, respectively. Group 5 yielded 238.2 PFU/mL (GMT) and 266.6 PFU/mL (GMT), and group 6 produced 21.5 PFU/mL (GMT) and 11.4 PFU/mL (GMT) for the first and second peaks, respectively. Overall, the plaque assay detected higher mean PFU titers for wtDENV1 and 4; this observation was consistent with RT-qPCR data.

**Table 11:** First-highest RNAemia sample's and second-highest RNAemia sample's plaque assay viremia titers.

Group	inoculum	First peak PFU/mL	Second peak PFU/mL
1	wtDENV1 (5 log_10_ PFU)	585	675
		1050	330
		100	300
2	wtDENV2 (5 log_10_ PFU)	no plaque	no plaque
		no plaque	no plaque
		no plaque	no plaque
3	wtDENV2 (3 log_10_ PFU)	no plaque	no plaque
		no plaque	no plaque
		no plaque	10
4	wtDENV3 (5 log_10_ PFU)	140	20
		120	30
		no plaque	no plaque
5	wtDENV4 (5 log_10_ PFU)	585	130
		70	270
		330	540
6	wtDENV4 (3 log_10_ PFU)	100	30
		10	10
		10	no plaque
7	TDV-AAAA (5, 5, 5, 5)	no plaque	no plaque
		no plaque	no plaque
		no plaque	10
8	TDV-AAAA (5, 3, 5, 3)	40	no plaque
		no plaque	no plaque
		no plaque	120
9	TDV-AAAA (5, 3, 3, 3)	no plaque	no plaque
		no plaque	no plaque
		no plaque	100
10	TDV-AAAB (5, 5, 5, 5)	no plaque	no plaque
		50	no plaque
		50	20
11	TDV-ABAB (5, 5, 5, 5)	30	no plaque
		70	no plaque
		80	no plaque
12	TDV-ABBB (5, 5, 5, 5)	90	no plaque
		170	no plaque
		no plaque	110

The first peak and the second peak were based on RT-qPCR data.

Although the serotypes of the plaques detected were not identified, all TDV-immunized monkeys (groups 7–12) had reduced infectious titers when compared with the single wt-challenged monkeys (groups 1–6), and the majority of TDV-immunized macaques developed measurable PFU titers at <2 log_10_ level. The monkeys in Group 7 TDV-AAAA (5, 5, 5, 5) showed no detectable infectious titers.

### 3.2 [Study 2]

#### 3.2.1 All individuals immunized with TDV-AAAA were protected against viremia following challenge

##### 3.2.1.1 Vaccination (pre-challenge) phase (Days 0–60)

The monkeys in Group 1 TDV (5, 5, 5, 5 log_10_ FFU) exhibited measurable viral loads for all DENV1–4 serotype components, and RNAemia peaked on day 1 with the hierarchy observed in the order of DENV4>DENV1>DENV2>DENV3 (Table 12). Positive titers were not detected in any monkeys on day 60, and they remained below LLOQ prior to the wtDENV challenge.

**Table 12:** Summary table of RNAemia levels of Study 2.

		Viral load in serum (log_10_ in genome copies/mL)											
		Vaccination phase						Challenge phase					
	Group	Day 0	Day 1	Day 2	Day 3	Day 4	Day 6	Day 60	Day 61	Day 62	Day 63	Day 64	Day 66
Against DENV1	(5, 5, 5, 5)	<2.65	4.79	4.43	3.78	2.98	2.67	<2.65	<2.65	<2.65	<2.65	<2.65	<2.65
	(4, 4, 4, 4)	<2.65	3.93	3.82	3.22	3.02	2.88	<2.65	<2.65	<2.65	<2.65	<2.65	<2.65
	(3, 3, 3, 3)	<2.65	3.08	3.25	3.09	3.18	3.16	<2.65	<2.65	<2.65	<2.65	<2.65	<2.65
	Vehicle	N/A	N/A	N/A	N/A	N/A	N/A	<2.65	6.80	6.48	5.24	3.80	3.59
Against DENV2	(5, 5, 5, 5)	<3.41	3.93	3.76	3.16	<3.11	<3.11	<3.11	<3.11	<3.11	<3.11	<3.11	<3.11
	(4, 4, 4, 4)	<3.11	3.39	3.71	3.14	<3.11	<3.11	<3.11	<3.11	<3.11	<3.11	<3.11	<3.11
	(3, 3, 3, 3)	<3.11	3.16	3.48	3.21	3.24	3.13	<3.11	<3.11	<3.11	<3.11	<3.11	<3.11
	Vehicle	N/A	N/A	N/A	N/A	N/A	N/A	<3.11	4.70	5.53	4.99	3.22	<3.11
Against DENV3	(5, 5, 5, 5)	<2.65	3.84	3.47	<2.65	<2.65	<2.65	<2.65	<2.65	<2.65	<2.65	<2.65	<2.65
	(4, 4, 4, 4)	<2.65	<2.65	<2.65	<2.65	<2.65	<2.65	<2.65	<2.65	<2.65	<2.65	<2.65	<2.65
	(3, 3, 3, 3)	<2.65	<2.65	<2.65	<2.65	<2.65	<2.65	<2.65	<2.65	<2.65	<2.65	<2.65	<2.65
	Vehicle	N/A	N/A	N/A	N/A	N/A	N/A	<2.65	4.00	3.44	3.16	<2.65	<2.65
Against DENV4	(5, 5, 5, 5)	<2.65	6.04	5.87	4.80	3.02	2.97	<2.65	<2.65	<2.65	<2.65	<2.65	<2.65
	(4, 4, 4, 4)	<2.65	4.84	4.98	4.56	3.86	2.97	<2.65	<2.65	<2.65	<2.65	<2.65	<2.65
	(3, 3, 3, 3)	<2.65	3.75	4.39	4.30	4.56	3.41	<2.65	<2.65	<2.65	<2.65	<2.65	<2.65
	Vehicle	N/A	N/A	N/A	N/A	N/A	N/A	<2.65	6.83	6.58	<2.65	<2.65	<2.65

<2.65 and <3.11 denotes that all three monkeys in the group had genome titer copies below LLOQ for the indicated day. The LLOQ was 900 GEq/mL for DENV1, 3, and 4 and 2,600 GEq/mL for DENV2. For the calculation of the GMT, data reported as “<900” were converted to “450” and data reported as “<2,600” were converted to “1,300.”

The monkeys in Group 2 TDV (4, 4, 4, 4 log_10_ FFU) produced measurable viral loads mostly for DENV1 and DENV4, and moderate levels (up to 2 × LLOQ) were observed for the DENV2 component. Similar to group 1, none of the monkeys yielded positive titers on day 60.

Group 3 TDV (3, 3, 3, 3 log_10_ FFU) monkeys exhibited reduced virus replication when compared with the higher dose groups (groups 1 and 2) as none of the monkeys developed detectable DENV2 and DENV3 RNAemia on day 1 (except two individuals for DENV2). None of the monkeys yielded a positive titer on days 14 and 60.

Group 4 control monkeys were administered with a vehicle on day 0 and served as DENV-naïve animals during the vaccination phase. All 12 monkeys in this group remained naïve by the challenge with wtDENV1–4 on day 60.

##### 3.2.1.2 Post-challenge phase (Days 60–74)

**Figure 3:**
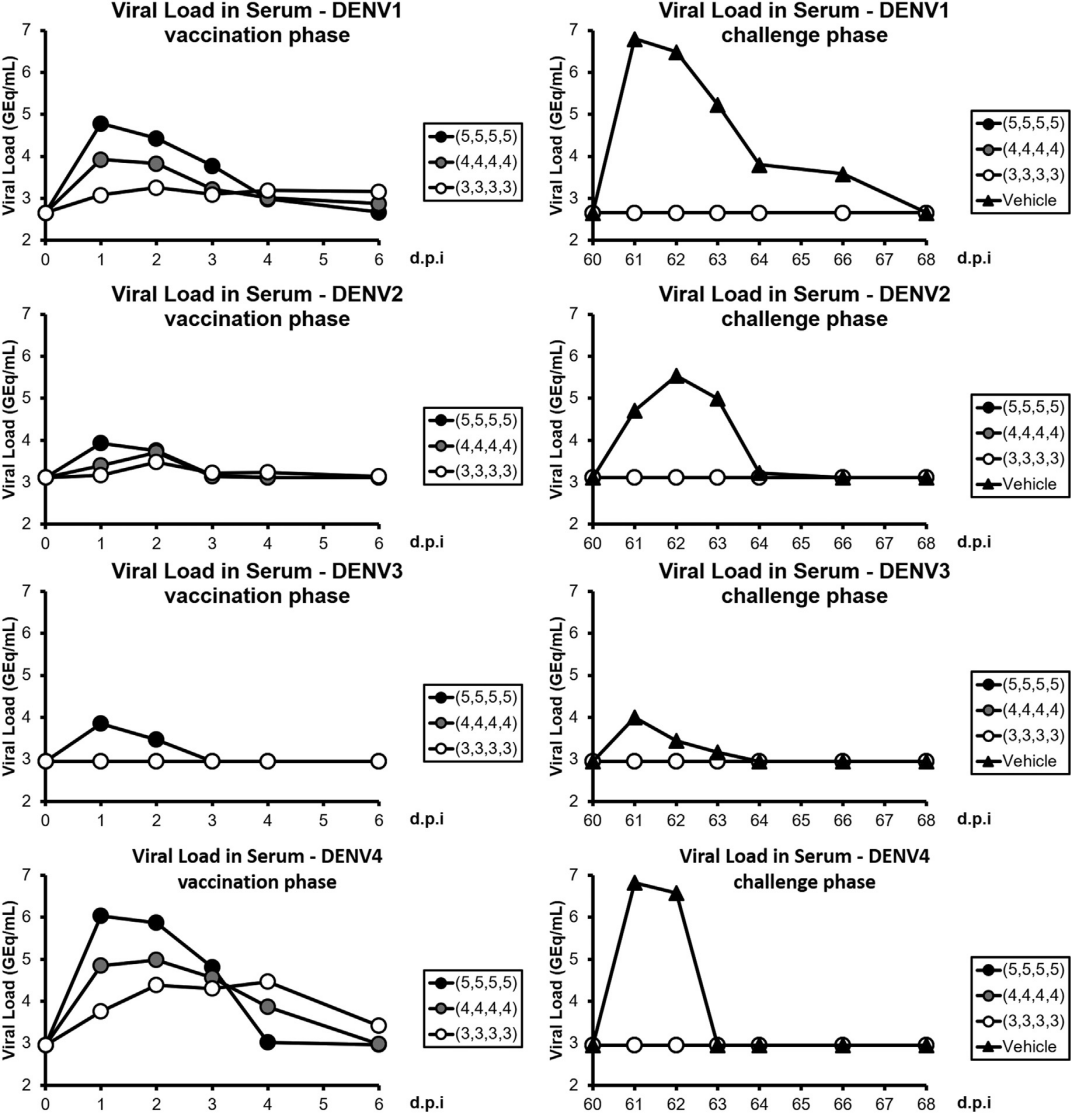
**The viral loads (GMT) in monkeys immunized with TDV candidates were elicited in a dose-dependent manner in the vaccination phase**. The viral load (genome copies/mL) was measured by RT-qPCR in serum samples collected prior to administration on Day 0 and on Days 1, 2, 3, 4, 6, 8, 10, 14, and 60 after administration. Only the day 8, 10, and 14 viral loads are shown in this figure. On day 60, all monkeys were inoculated with wtDENVs, and those in the vehicle group elicited higher viral loads than those in the TDV vaccination phase. Vaccinated monkeys (group 1, 2, and 3) were completely protected after the challenge.

On day 60, each TDV group was further divided into four subgroups for a challenge with wtDENV1, 2, 3, or 4, with challenge doses of 1 × 10^5^, 1 × 10^5^, 1 × 10^6^, or 1 × 10^5^ PFU/animal, respectively, for assessment of vaccine protective efficacy. Vehicle control (group 4) animals received the same challenge dose as described in Table 4 (N = 3 per serotype). Markedly, regardless of the vaccine dose, TDV vaccinated macaques in groups 1–3 were completely protected against wtDENV1–4 RNAemia, with viral loads below LLOQ (Figure 3).

#### 3.2.2 All individuals immunized with three TDV-AAAA dose formulations seroconverted for all four serotypes

**Figure 4:**
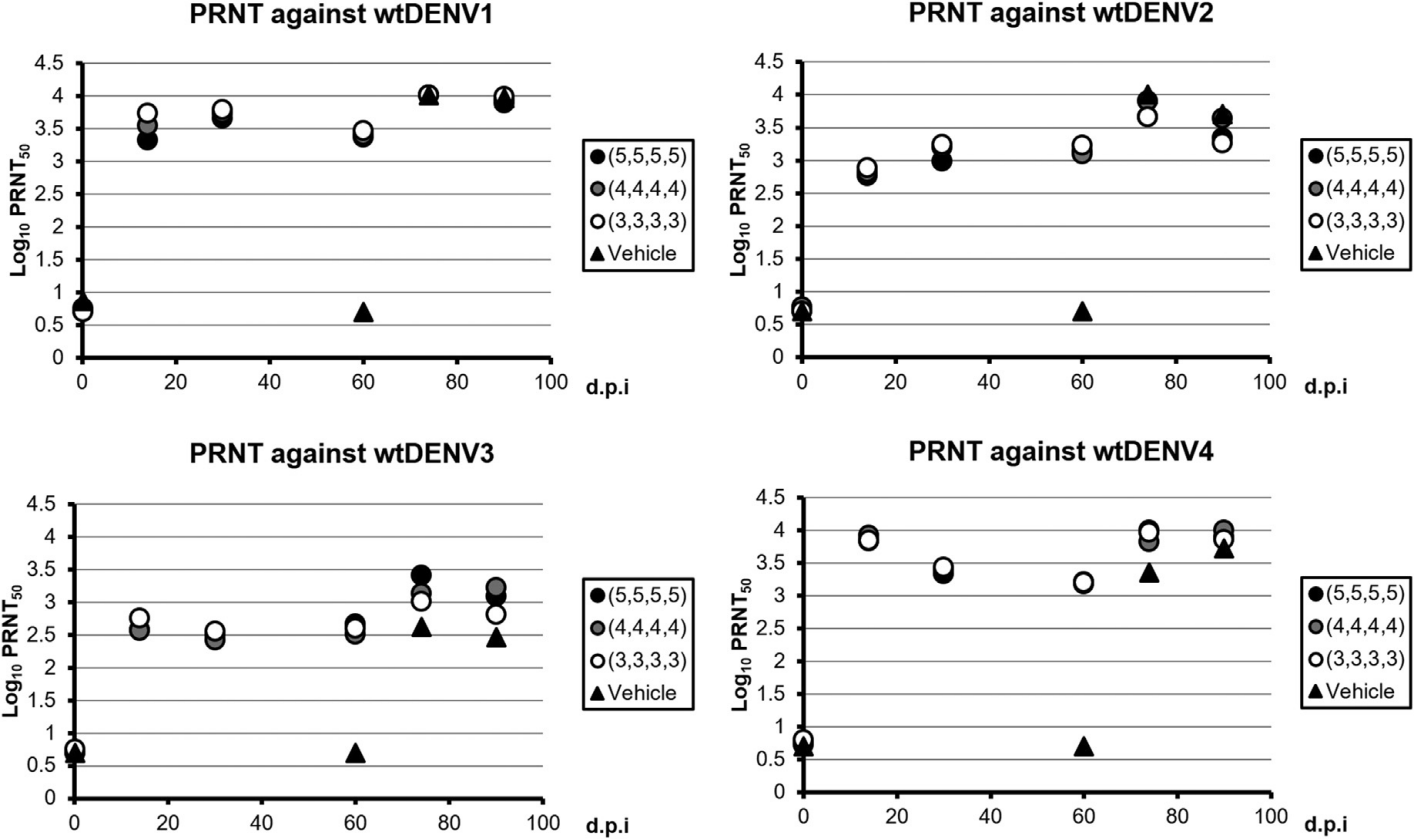
**KD-382 elicited sterile NAb responses**. A PRNT assay against the wt parental DENV1–4 virus was conducted in serum samples collected prior to administration on Day 0 and on days 14, 30, 60, 74, and 90 after administration. The immune responses to TDVs were elicited in a dose-independent manner. For the calculation of the GMT, data reported as “<10” were converted to “5” and data reported as “≥10,240” were converted to “10,240”.

We selected the TDV candidate formulation (TDV-AAAA) for further evaluation based on the following features in study 1: 1) Seroconverted for all four serotypes at 14 days after administration; 2) Induced a balanced NAb for all four serotypes; and 3) Displayed a low RNAemia and no viremia (i.e., no measurable infectious virus) after administration. All monkeys immunized in Group 1 TDV (5, 5, 5, 5), Group 2 TDV (4, 4, 4, 4), and group 3 TDV (3, 3, 3, 3) seroconverted to each of the four DENV components as early as day 14. Dose responses were not observed, as there were no significant differences in NAb titers between the TDV groups (groups 1–3) (p < 0.05). The DENV3 NAb titer was constant in all three groups. The DENV4 NAb titer declined twofold to threefold in all three dose groups when compared with the titers between day 14 and day 30, but the titer remained >3.0 log_10_ GMT. The NAb levels against each serotype were comparable between groups 1–3, thus immune responses to TDVs were elicited in a dose-independent manner. On day 74, 14 days after the wtDENV challenge, all monkeys in TDV groups 1–3 exhibited elevated NAbs against DENV1–4. On day 90, all of the TDV macaques in groups 1–3 maintained high NAb levels. Similar to day 74, the day 90 NAb levels against each serotype did not vary between dose groups and log_10_ GMT to each serotype in the three TDV groups. All of the group 4 vehicle control animals that received a single wtDENV on day 60 (N = 3 per serotype) seroconverted and developed high NAbs against the corresponding virus (Tables 13 and 14, Figure 4).

**Table 13:** Summary table of NAb levels following TDV-AAAA immunization.

		Geometric mean serum NAb titer (reciprocal dilution log_10_ PRNT_50_) against:															
		Day 0				Day 14				Day 30				Day 60			
Group	Inoculum	DENV1	DENV2	DENV3	DENV4	DENV1	DENV2	DENV3	DENV4	DENV1	DENV2	DENV3	DENV4	DENV1	DENV2	DENV3	DENV4
1	TDV-AAAA (5, 5, 5, 5)	0.721	0.699	0.699	0.723	3.321	2.761	2.573	3.838	3.655	2.985	2.500	3.336	3.368	3.141	2.662	3.179
2	TDV-AAAA (4, 4, 4, 4)	0.756	0.769	0.699	0.754	3.544	2.821	2.563	3.925	2.729	3.200	2.416	3.396	3.401	3.102	2.513	3.211
3	TDV-AAAA (3, 3, 3, 3)	0.699	0.699	0.755	0.801	3.726	2.887	2.751	3.847	3.785	3.241	2.558	3.434	3.468	3.226	2.594	3.200
4	Vehicle	0.867	0.699	0.699	0.699	N/A	N/A	N/A	N/A	N/A	N/A	N/A	N/A	0.699	0.699	0.699	0.699

For the calculation of the GMT, data reported as “<10” were converted to “5,” and data reported as “≥10,240” were converted to “10,240.”

**Table 14:** Summary table of NAb levels following wtDENVs challenge.

		Geometric mean serum NAb titer (reciprocal dilution log_10_ PRNT_50_) against:							
		Day 74				Day 90			
Group	Inoculum	DENV1	DENV2	DENV3	DENV4	DENV1	DENV2	DENV3	DENV4
1	TDV-AAAA (5, 5, 5, 5)	4.010	3.659	3.411	4.006	3.882	3.344	3.082	3.905
2	TDV-AAAA (4, 4, 4, 4)	4.010	3.909	3.129	3.824	3.935	3.635	3.219	4.007
3	TDV-AAAA (3, 3, 3, 3)	4.010	3.666	3.015	3.964	3.986	3.264	2.819	3.855
4	Vehicle	4.010	4.010	2.619	3.362	3.965	3.703	2.465	3.728

The wtDENVs subcultured parental clinical isolates with Vero cells several times were used as the challenge viruses. Animals in each group were divided into four parts with equal number (n = 6 for Groups 1 to 3, n = 3 for Group 4) and challenged with one of four wtDENV serotypes as shown in Table 4. In each corresponding part, the GMT against the same serotype used for challenge were calculated. For the calculation of the GMT, data reported as “<10” were converted to “5,” and data reported as “≥10,240” were converted to “10,240.”

#### 3.2.3 Genome analyses identified potential attenuating mutations

TDV-AAAA induced a lower level of RNAemia than wt DENV. To further explore this attenuated phenotype at the genomic level, deep sequencing of both parental viruses and vaccine seed viruses of candidate A was conducted. Amino acid changes were identified between the parental viruses and vaccine seed viruses of each serotype (Table 15).

**Table 15:** Amino acid changes in vaccine seed viruses of candidate A from corresponding parental viruses.

Region	Position	Parental virus	Vaccine seed virus
**Serotype 1**			

pre	85	Glu/Lys	Glu
E	204	Lys	Glu/Lys
E	205	Glu	Lys
E	206	Lys	Arg/Lys
E	290	Lys	Arg
NS1	224	Val/Ile	Ile
NS3	188	Asn	Lys
NS4B	109	Ile/Thr	Thr
NS4B	113	Leu	Leu/Phe
NS4B	116	Val/Ala	Val
NS4B	120	Ala	Thr/Ala

**Serotype 2**			

pre	29	Asp	Asn
E	118	Lys/Met	Met
E	120	Thr	Lys
E	204	Lys/Arg	Lys
NS1	327	Asp	Asn
NS2A	181	Leu	Phe
NS2B	116	Val	Val/Ala
NS3	179	Glu	Lys
NS4A	97	Tyr	Tyr/Cys
NS4B	104	Pro	Pro/Leu
NS4B	108	Ile	Thr
NS5	337	Thr	Thr/Met
NS5	529	Ala	Ala/Thr

**Serotype 3**			

pre	82	Gln/His	Gln
M	4	Ile	Leu
E	205	Leu/Met	Met
E	302	Ser	Gly
E	391	Lys/Arg	Lys
E	407	Ala	Val
E	484	Thr	Ile/Thr
NS2A	86	Phe	Leu
NS2A	112	Ala	Thr
NS2B	58	Glu/Asp	Asp
NS3	90	Gln	Lys
NS4B	197	Val/Ala	Ala
NS5	404	Ala/Thr	Thr

**Serotype 4**			

NS1	253	Gln	His
NS2B	85	Ser/Phe	Ser
NS4A	95	Leu	Phe
NS4A	125	Lys/Arg	Lys
NS4B	102	Ile/Thr	Thr
NS4B	112	Phe/Leu	Ser
NS5	390	Asn/Lys	Asn

## 4 Discussion

Many infectious diseases have been controlled by vaccine development [29]. The development of the dengue vaccine together with vaccines for malaria and AIDS has been a long-standing dream of scientists and a critical milestone of public health. Now, as described in Materials and Methods, we tried to generate a new set of TDVs based on the precedent TDV (Mahidol TDV) mainly by the improvement of the DENV3 strain; we evaluated the potential of the new candidates in non-human-primate (NHP) models.

Based on the findings of study 1, we selected the TDV candidate combination (TDV-AAAA) for further evaluation. In order to evaluate the dose response regarding the immunogenicity and protective efficacy in the NHP model, study 2 was conducted using different doses of TDV-AAAA consisting of either all 3, 4, or 5 logs FFU/dose under the GLP compliant condition. Although no RNAemia was detected after the TDV administration in study 1, dose-dependent RNAemias for all four serotypes were detected in study 2, even though they were lower than those of the corresponding wt strains. This is probably because the extraction process was more optimized by changing the RNA extraction kit from RNA Stat-60 RNA Extraction Reagent in study 1 to QIAamp viral RNA mini kit in study 2. As for the reproducibility of results obtained in study 1, the tetravalent seroconversion rate reached 100% on day 14 in all three different dose formulations. In study 2, the GMT of the NAb titers against DENV1, 2, 3, and 4 were balanced at 60 days after the single administration of TDV-AAAA and before the challenge; however, the GMT for DENV3 was slightly lower than the others and there was no dose dependency. This phenomenon also occurred in previous monkey studies with monovalent vaccines (NAb titer (GMT, n = 6) induced by mono-DENV: DENV1: 3.81; DENV2: 3.66; DENV3: 3.16; DENV4: 4.01, respectively). All animals administered with a single dose of the tetravalent vaccine were completely protected from viremia after being challenged with the wt strain for all four serotypes.

In both studies 1 and 2, viremia derived from the vaccine strains was detected, with the exception of DENV3; however, the magnitude was quite a bit lower than that of the wt strains. For DENV3, the RNAemia induced by both the vaccine and wt viruses was very weak and can not compare them. In study 1, a viral load of the monkey serum inoculated with wtDENV3 recovered a considerable number of plaques at both PCR Peaks 1 and 2. From the serum derived from monkeys administered with vaccine formulations other than TDV-AAAA (containing all five log_10_ viruses), a considerable number of plaques were also recovered. Although the serotypes of the plaques recovered from the vaccinated monkeys were not identified, they were assumed to be serotypes other than DENV3 based on the PCR results. The evidence of viremia after administration of TDV-AAAA and corresponding parental wt viruses indicates that a proliferation of the vaccine viruses of TDV-AAAA in vivo would be limited; therefore, the vaccine strains can be considered to have a sufficient safety profile (i.e., attenuation) for further evaluation in human clinical studies. There is a risk of a virulent revertant virus emerging from a live attenuated virus. In this study, the vaccine seed viruses were confirmed to have at least three complete amino acid changes when compared with the parental viruses, which could reduce the chance of revertant emergence. Further study to identify the attenuating mutations and evaluate the genetic stability is required.

We evaluated several TDVs with different combinations of viruses in monkeys, and the most favorable combination (TDV-AAAA, 5, 5, 5, 5), which could induce a well-balanced antibody response and lower viremia profiles compared with the corresponding parental wtDENVs, was selected for further evaluation and named it “KD-382”. In the dose selection NHP study of KD-382, no dose response in the immune response and protective efficacy was found in the range of 3–5 log_10_ FFU/dose. Monkeys given a single dose of KD-382 were completely protected from RNAemia upon s.c. challenge with wtDENV virus. The result satisfy one of the requirements for pre-clinical testing in the development of dengue vaccines [30]. Therefore, we concluded that KD-382 has potential that could be further evaluated in a Phase 1 study with a dose escalation method.

Before proceeding to clinical trials, further NHP studies are planned to clear the following issues:

✓ number of doses required,
✓ antibody persistence after a single dose,
✓ efficacy against a challenge more than six months after single-dose immunization,
✓ immunogenicity in individuals previously primed with one of each serotype of the wt virus.

## Declarations

### Author contribution statement

Masaya Yoshimura, Shota Takagi: Performed the experiments; Analyzed and interpreted the data; Contributed reagents, materials, analysis tools or data; Wrote the paper.

Yasuhiko Shinmura: Conceived and designed the experiments; Performed the experiments; Analyzed and interpreted the data; Contributed reagents, materials, analysis tools or data; Wrote the paper.

Kazuhisa Kameyama, Sutee Yoksan: Analyzed and interpreted the data.

Kengo Sonoda: Conceived and designed the experiments.

Fusataka Koide: Performed the experiments; Contributed reagents, materials, analysis tools or data.

Kazuhiko Kimachi: Conceived and designed the experiments; Wrote the paper.

### Funding statement

This work was supported by the Global Health Innovative Technology (10.13039/501100013996GHIT) Fund (RFP2014-001) and KM Biologics Co., Ltd.

### Competing interest statement

The authors declare the following conflict of interests: Masaya Yoshimura, Yasuhiko Shinmura, Shota Takagi, Kazuhisa Kameyama, Kengo Sonoda, Kazuhiko Kimachi; full-time employees of KM BiologicsCo., Ltd.; Fusataka Koide; full-time employee of Southern Research.

### Additional information

No additional information is available for this paper.
